# The fibrogenic actions of lung fibroblast-derived urokinase: a potential drug target in IPF

**DOI:** 10.1038/srep41770

**Published:** 2017-01-31

**Authors:** Michael Schuliga, Jade Jaffar, Trudi Harris, Darryl A Knight, Glen Westall, Alastair G Stewart

**Affiliations:** 1 Lung Health Research Centre, Department of Pharmacology and Therapeutics, University of Melbourne, Parkville, Victoria, Australia; 2 School of Biomedical Sciences and Pharmacy, University of Newcastle, Callaghan, New South Wales, Australia; 3 Priority Research Centre for Healthy Lungs, Hunter Medical Research Institute, New Lambton Heights, New South Wales, Australia; 4 Allergy, Immunology and Respiratory Medicine, Alfred Hospital, Prahran, Victoria, Australia; 5 Department of Anesthesiology, Pharmacology and Therapeutics, University of British Columbia, Canada

## Abstract

The role of urokinase plasminogen activator (uPA) in idiopathic pulmonary fibrosis (IPF) remains unclear. uPA-generated plasmin has potent fibrogenic actions involving protease activated receptor-1 (PAR-1) and interleukin-6 (IL-6). Here we characterize uPA distribution or levels in lung tissue and sera from IPF patients to establish the mechanism of its fibrogenic actions on lung fibroblasts (LFs). uPA immunoreactivity was detected in regions of fibrosis including fibroblasts of lung tissue from IPF patients (n = 7). Serum uPA levels and activity were also higher in IPF patients (n = 18) than controls (n = 18) (P < 0.05), being negatively correlated with lung function as measured by forced vital capacity (FVC) %predicted (P < 0.05). The culture supernatants of LFs from IPF patients, as compared to controls, showed an increase in plasmin activity after plasminogen incubation (5–15 μg/mL), corresponding with increased levels of uPA and IL-6 (n = 5–6, P < 0.05). Plasminogen-induced increases in plasmin activity and IL-6 levels were attenuated by reducing uPA and/or PAR-1 expression by RNA*i*. Plasmin(ogen)-induced mitogenesis was also attenuated by targeting uPA, PAR-1 or IL-6. Our data shows uPA is formed in active regions of fibrosis in IPF lung and contributes to LF plasmin generation, IL-6 production and proliferation. Urokinase is a potential target for the treatment of lung fibrosis.

Idiopathic pulmonary fibrosis (IPF) is characterized by a progressive decline in lung function, usually leading to death within 5 years of diagnosis. IPF, considered to be the end result of a dysregulated repair response of epithelial/fibroblast origin, features excessive numbers of fibroblasts and collagen accumulation in lung parenchyma, causing structural defects that impair gas exchange^1^. Glucocorticoids show no appreciable effect on clinical outcome^2^. Until recently, proven treatment options were limited to lung transplantation. Pivotal clinical trials of pirfenidone and nintedanib have shown that each of these drugs reduces the rate of decline in lung function and increases progression free survival in IPF, albeit with modest effect and significant side effects^3^,^4^. These positive, though limited developments, have encouraged renewed interest in identification of alternative and complementary drug targets.

Urokinase plasminogen activator (uPA) has fibrogenic actions^5^, albeit its pathological role in IPF is not entirely understood. uPA-generated plasmin can evoke concomitant collagen proteolysis and (trans)activation of protease activated receptor-1 (PAR-1), integrins and transforming growth factor-β receptor (TGFβR), indirectly implicating this system in IPF pathology^6^. However, plasmin-mediated fibrinolysis is suppressed in IPF, contributing to the accumulation of airspace fibrin^7^,^8^. Suppressed fibrinolysis corresponds with lower levels of uPA detected in the bronchoalveolar lavage fluid (BALF) of IPF patients, conversely with higher levels of plasminogen activator inhibitor-1 (PAI-1)^9^,^10^. Consequently, uPA has been considered to be protective, rather than detrimental in IPF. However, fibrinolysis is the physiological role of tissue-type plasminogen activator (tPA), which has fibrin specificity, unlike uPA. Nevertheless, intranasal administration of supra-physiological concentrations of uPA or the overexpression of uPA in bronchial Clara cells of the epithelium augment airspace fibrinolysis, being protective in experimental lung injury^11^,^12^. Still, the therapeutic effects of ectopic uPA do not exclude potential pathological contributions of endogenous uPA formed in the lung interstitium. In IPF, interstitial uPA-generated plasmin is expected to have potent fibrogenic actions involving PAR-1 and other mechanisms^13^. To our knowledge, no study has explored in depth the distribution of uPA in lung tissue of IPF patients, nor examined the potential role of interstitial plasmin in IPF. We hypothesise that in IPF, the pericellular balance between uPA and PAI-1 levels in the lung interstitium are disturbed in favour of localized increases in plasmin. In support, bleomycin-induced lung injury increases localized plasminogen activation in the parenchymal tissue of mice, despite fibrinolysis in the airspaces being suppressed^14^.

Interleukin-6 (IL-6) is a multifunctional cytokine produced by cell types with important roles in IPF including lung fibroblasts and macrophages^15^,^16^. IL-6 expression is increased in response to a number of fibrogenic stimuli, including proteases that activate PAR-1^17^,^18^. Serum and BALF levels of IL-6 are increased in interstitial lung diseases (ILDs), being predictive of lung function decline and mortality^19^. Other members of the IL-6 cytokine family, that signal through glycoprotein 130 (gp130), including oncostatin M (OSM), are also implicated in lung fibrosis, including IPF^20^. Inhibiting IL-6 activity is protective in pre-clinical models of lung injury and fibrosis^21^,^22^. Lung fibroblasts exposed to lung edema fluid from patients with early stage acute respiratory distress syndrome (ARDS) produce increased amounts of IL-6, which in turn stimulates fibroblast activation and proliferation in an autocrine manner via gp130 signaling^15^. Furthermore, dysregulated gp130 signaling is also implicated in IPF^23^.

Our hypothesis that interstitial uPA has a detrimental role in IPF is novel, accounting for nuances in functional and spatial compartmentalisation of plasminogen activation in lung pathology. In this study, the levels and/or distribution of uPA in lung tissue and serum from IPF patients was for the first time investigated, as was the role of uPA-generated plasmin in mediating fibrogenic actions of lung fibroblasts. We provide clear evidence that uPA expression is associated with regions of active fibrosis in IPF, and that levels and activity of uPA in serum of IPF patients are increased. Furthermore, uPA-dependent plasminogen-evoked IL-6 production and subsequent proliferation was shown to be greater in lung fibroblast cultures from IPF patients. These observations suggest that interstitial uPA contributes to lung fibrosis and may be a useful target in the treatment of lung disease.

## Results

### uPA is expressed in fibrotic lung tissue from IPF patients

**Figure 1:**
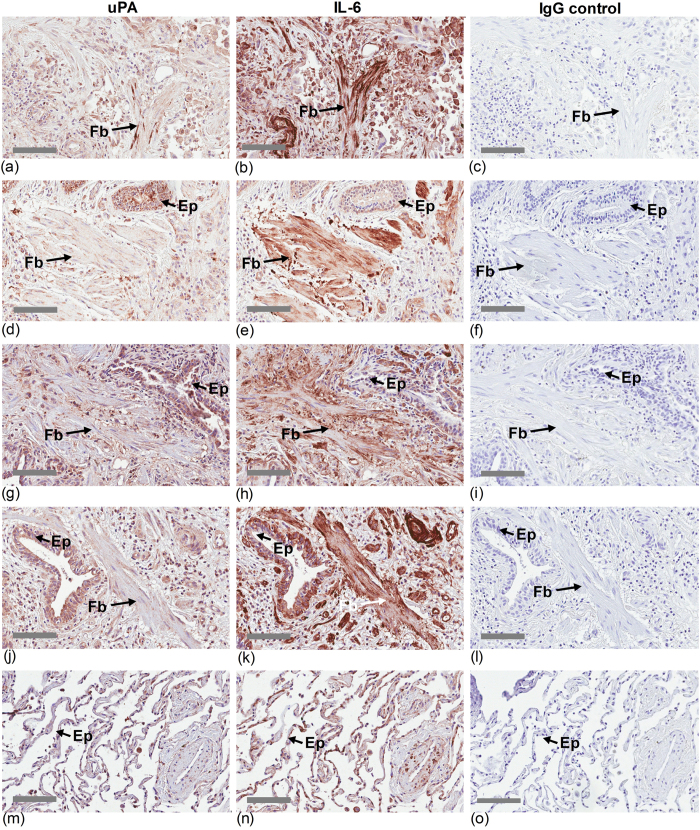
uPA and IL-6 immunoreactive cells are detected in fibrotic lung tissue of IPF patients. Serial sections of parenchymal tissue from separate IPF patients, (**a–c**) ALF016, (**d–f**) ALF023, (**g–i**) ALF028 and (**j–l**) ALF029, as well as a control donor, (**m–o**) ALF012. Sections were immunostained for uPA (left panel) or IL-6 (middle panel). The negative rabbit IgG control of the lung sections is also shown (right panel). The sections from IPF patients show uPA and IL-6 staining (brown) in fibroblast (Fb) and epithelial (Ep) cells. The scale bar in images are 100 micron.

**Figure 2:**
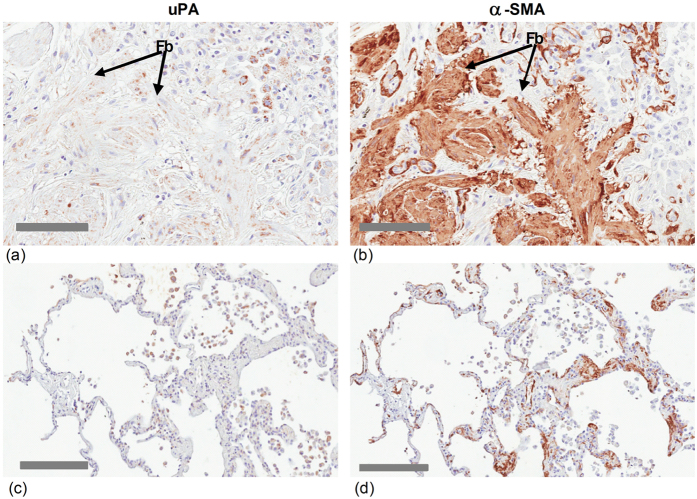
uPA and α-SMA immunoreactivity overlap in fibrotic lung tissue of IPF patients. Sequential serial lung tissue sections from either (**a**–**b**) IPF patient (ALF027) or (**c**–**d**) control (ALF024) stained for (**a,c**) uPA or (**b,d**) α-smooth muscle actin (α-SMA). The scale bars in images are (**a–b**) 200 or (**c–d**) 100 micron.

The presence and distribution of uPA and IL-6 in lung tissue from IPF patients was identified by immunohistochemistry (IHC). uPA immunoreactivity was readily detected in the consolidated regions of lung tissue from IPF patients (n = 7) (Figs 1, 2 & Supplement, Fig. S1). These regions of fibrosis are heavily stained for collagen and α-smooth muscle actin (α-SMA), which is expressed in mesenchymal cells (Supplement, Fig. S1). Well-differentiated uPA staining was observed associated with both fibroblast and epithelial cells in fibrotic lung, which also stained intensely for IL-6 (Fig. 1). uPA immunoreactivity also overlapped with α-SMA staining in cells of serial sections of lung tissue from IPF patients (Fig. 2). uPA was also detected in the alveolar epithelium of lung tissue of controls (n = 4) (Figs 1 and 2, Supplement, Fig. S2).

### Serum uPA levels and activity are increased in IPF

**Figure 3:**
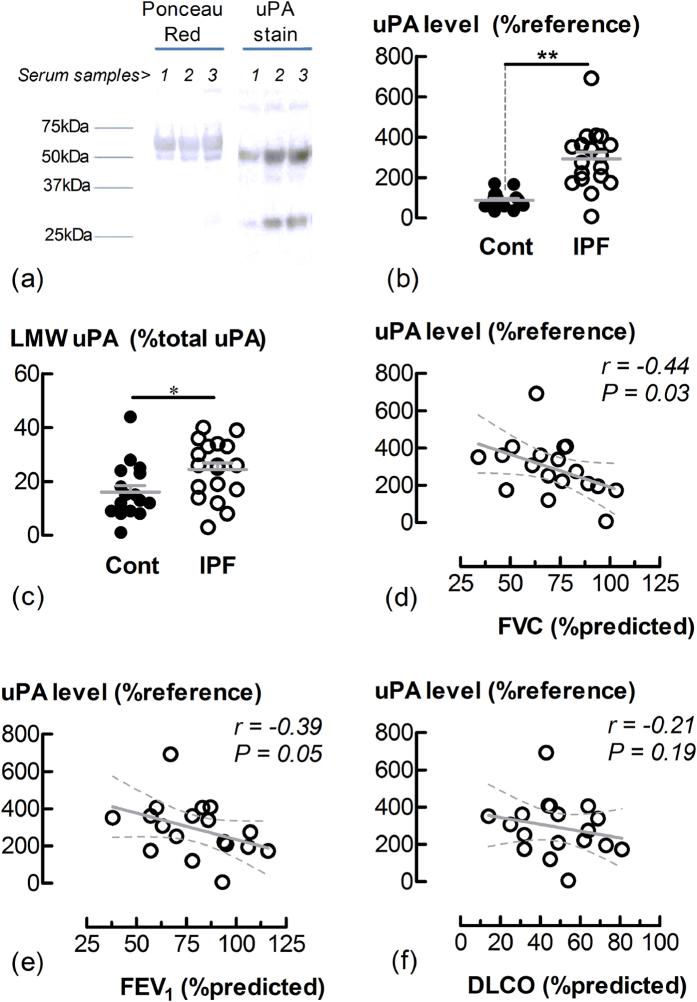
Serum levels of uPA are increased in IPF. Sera (10 μg protein) from IPF or control donors in parallel were resolved by SDS-PAGE and immunoblotted for uPA. (**a**) Blots of serum samples showing overall protein loading by Ponceau red staining and both the high and low molecular weight forms of uPA (~54 and 31 kDa, respectively). Lane 1 is serum from a control donor, whereas lanes 2 and 3 are sera from separate IPF donors. (**b**) The relative levels of total uPA (high and low MW forms combined) in serum based on densitometry analysis. **P < 0.01 (n = 18 IPF and n = 18 non-IPF). (**c**) The percentage of low MW uPA to total uPA. *P < 0.05. (**d**–**f**) The relationships between uPA levels of IPF patients and lung function as measured by the % predicted values of FVC, FEV_1_ and DLCO. The solid line is the regression line, whereas the dotted lines represent the 95% confidence intervals. Data (**d**–**f**) were analyzed using the Pearson’s correlation test, with r and P (one-tailed) values provided.

To investigate whether uPA serum levels are altered in IPF, samples were resolved by SDS-PAGE and immunoblotted for uPA. The high and low MW forms of uPA (~54 and 31 kDa, respectively) were detected in the serum of both IPF patients and controls (Fig. 3a). The sera of IPF patients had higher immunoreactive uPA levels than those of the controls (P < 0.01) (Fig. 3b), with the ratio of low MW to total uPA being higher in disease (P < 0.05) (Fig. 3c). There was a negative correlation with a Pearson correlation coefficient of −0.44 between uPA levels and lung function as measured by forced vital capacity (FVC) %predicted for IPF patients (P < 0.05) (Fig. 3d). The coefficients for the %predicted values of forced expiratory volume (FEV_1_) and diffusing capacity of lung for carbon monoxide (DLCO) were −0.39 (P = 0.054) and −0.22 (P = 0.19) respectively (Fig. 3e–f). uPA enzyme activity, measured using a fluorogenic substrate, was also higher in sera for IPF patients, and negatively correlated with lung function, as measured by FVC %predicted (Supplement, Fig. S3). There was no correlation between uPA and age for either the IPF or control groups (Supplement, Fig. S4). Furthermore, serum PAI-1 levels were not elevated in IPF (Supplement, Fig. S5).

### Plasminogen stimulates increased IL-6 production by LFs

**Figure 4:**
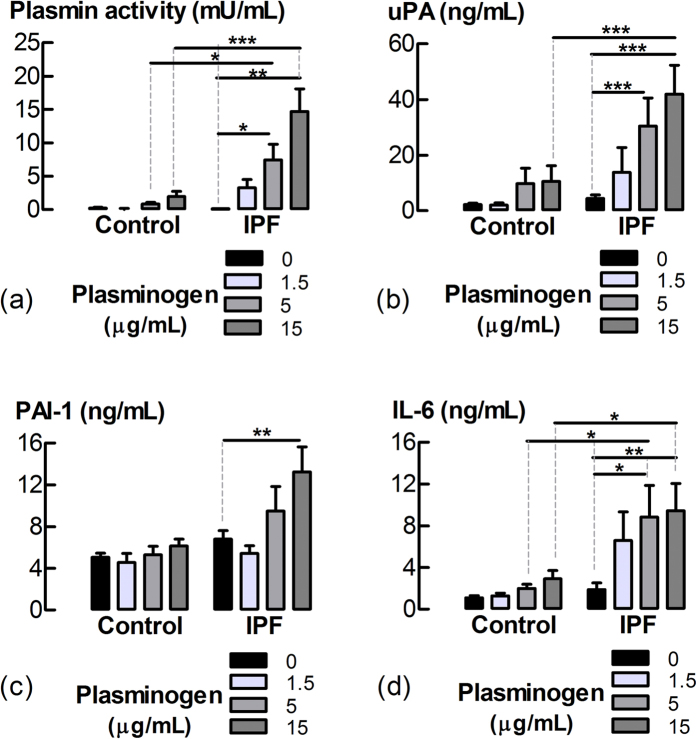
Plasminogen stimulates increased IL-6 and uPA production by LFs in association with greater plasmin activity. Levels of (**a**) plasmin activity, (**b**) uPA, (**c**) PAI-1 and (**d**) IL-6 in the media conditioned by LFs from IPF (n = 6) and control (n = 5–6) donors incubated with plasminogen for 24 h. *P < 0.05, **P < 0.01.

The effect of lung fibroblast (LF) plasminogen activation on IL-6 production was examined. Incubation with plasminogen (5 and 15 μg/mL) for 24 h resulted in larger increases in plasmin and IL-6 levels in media conditioned by LFs of IPF patients than in those of the controls (Fig. 4). Interestingly, plasminogen (5 and 15 μg/mL) increased uPA production by IPF-derived LFs, but not controls. Levels of PAI-1 in cultures of LFs were unchanged, except at the highest concentration of plasminogen examined, which stimulated a modest increase and only in IPF cell lines.

### uPA and PAR-1 mediate plasminogen-evoked IL-6 production

**Figure 5:**
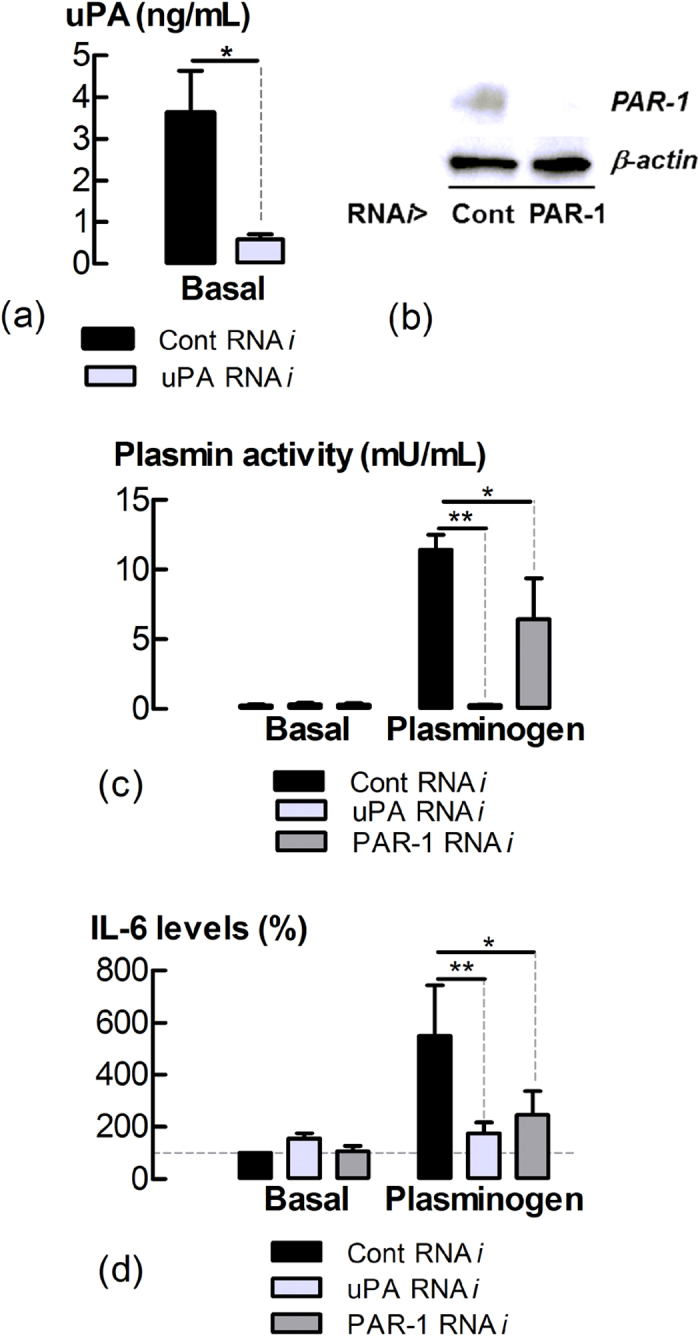
uPA and PAR-1 regulate the conversion of plasminogen into plasmin and subsequent IL-6 production. (**a**) Levels of uPA in cultures of RNA*i*-transfected LFs. Data analyzed by student’s t test. (**b**) An immunoblot which shows PAR-1 protein knock-down in cell lysates by RNA*i* transfection (representative of 3 experiments). Supernatant levels of (**c**) plasmin activity and (**d**) IL-6 in cell cultures following RNA*i* transfection and 24 h plasminogen (15 μg/mL) incubation. *P < 0.05, **P < 0.01 (n = 3–5).

PAR-1 is a known receptor for plasmin. The involvement of uPA and PAR-1 in regulating LF generation of plasmin and IL-6 production was examined. Decreasing uPA and PAR-1 expression (Supplement, Fig. S6) and protein (Fig. 5 a-b) by RNA*i* transfection decreased plasminogen-associated plasmin activity and IL-6 levels in LF conditioned media (Fig. 5c-d). UK122 (10 μM), a pharmacological uPA inhibitor^24^ also attenuated plasminogen-evoked increases in plasmin production (P < 0.05, n = 6) (Supplement, Fig. S7).

### Plasmin-stimulated IL-6 production is similar for LFs of IPF and controls

To ascertain whether plasmin mediates the effects of plasminogen on IL-6 production, LFs were incubated with concentrations of plasmin spanning those detected in the supernatants of plasminogen-incubated cells (1.5–15 mU/mL). The magnitude of plasmin-induced increases in IL-6 levels was unrelated to donor IPF status (Table 1). Interestingly, plasmin at 15 mU/mL stimulated uPA production, this effect being greater for LFs from IPF patients than controls (P < 0.05, n = 5–6). Targeting PAR-1, using RNA*i* or neutralizing IgG attenuated plasmin-evoked increases in IL-6 levels (Supplement, Fig. S8). The effects of plasmin were also attenuated by PD98059 (ERK inhibitor) and SB203580 (p38 MAPK inhibitor), but not the PI3K/Akt inhibitor, LY294002 (Supplement, Fig. S9).

### IL-6 mediates the proliferative actions of plasminogen

**Figure 6:**
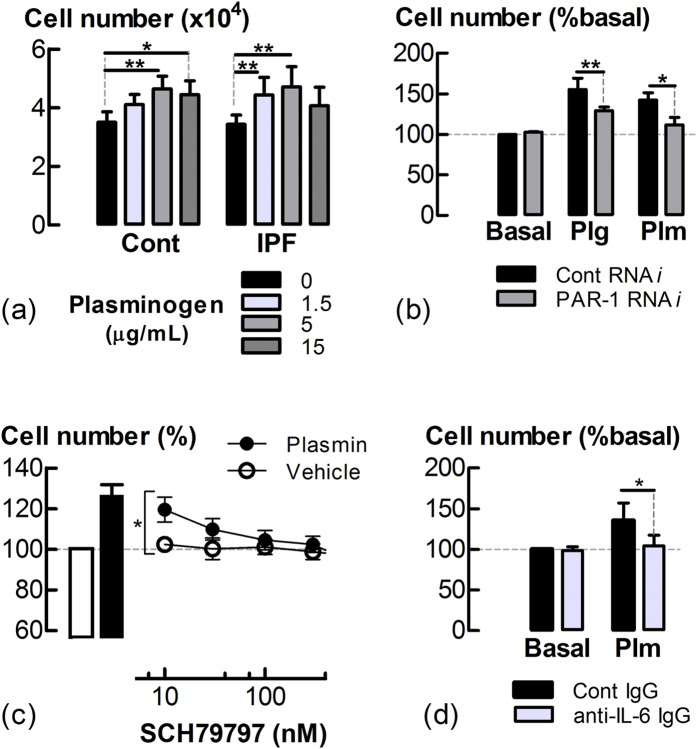
Plasmin(ogen) stimulates LF proliferation in an IL-6-dependent manner. (**a**) Attached cell number following incubation with plasminogen (1.5–15 μg/mL) for 48 h (n = 5 control donors and n = 7 IPF donors). (**b**) Attached cell number following RNA*i* transfection and subsequent plasminogen (Plg, 15 μg/mL) or plasmin (Plm, 5 mU/mL) incubation. (**c**) Effect of SCH79797, a PAR-1 inhibitor, on plasmin-stimulated increases in cell number. (**d**) Cell number following incubation with plasmin and anti-IL-6 or control IgG (2 μg/mL) for 48 h. *P < 0.05, **P < 0.01, (n = 3–7).

Incubation with plasminogen (1.5–15 μg/mL) for 48 h elicited a larger increase in cell number in both IPF and control LF cell cultures (Fig. 6a). There was an apparent decrease in the number of attached cells at plasminogen concentrations of or more than 15 μg/mL, regardless of donor status. To examine whether such decreases were a consequence of cell detachment, the effect of a broader range of plasminogen concentrations on both the number of attached and detached cells in culture was investigated. Plasminogen (50 μg/mL) increased the number of detached LFs in culture, corresponding to a decrease in the number of adherent cells (Supplement, Fig. S10). Exogenous plasmin 50 mU/mL also increased the number of detached cells (Supplement, Fig. S10). At lower concentrations (1.5–5 mU/mL), plasmin increased the adherent cell number. Plasmin(ogen) induced mitogenic responses were attenuated by PAR-1 RNA*i* (Fig. 6b), as was the associated increased expression of the cell cycle regulatory protein, cyclin D1 (CCND1) (Supplement, Fig. S11). Furthermore, plasmin-stimulated increases in cell number were attenuated by the PAR-1 antagonist, SCH79797^18^ (Fig. 6c) or the function neutralising anti-IL-6 IgG (Fig. 6d).

## Discussion

Our finding of increased tissue levels of uPA in IPF patients is consistent with a contribution of interstitial plasmin activity to pulmonary fibrosis. We now show for the first time that uPA mediates plasminogen activation by human lung fibroblasts, in association with increased IL-6 production and proliferation. These fibrogenic responses evoked by plasminogen involve increased plasmin generation by lung fibroblasts. The activity of the plasmin generation pathway is amplified in lung fibroblasts from IPF patients. Genetic and pharmacological evidence indicates that PAR-1 contributes to plasminogen-evoked fibrogenesis, acting as a plasmin receptor. Furthermore, we establish the novel observation that serum levels of uPA are higher in the serum of IPF patients, and importantly show an inverse correlation with lung function.

Vascular leak leads to extravasation of plasma proteins into parenchymal tissue following injury and in disease^25^. Thus, lung fibroblasts would be exposed to plasma-derived plasminogen, with concentrations spiking during acute inflammation/exacerbation. We detected uPA in fibrotic lung tissue of IPF patients, associated with both fibroblasts and epithelial cells, consistent with reported uPA and uPAR expression within fibroblasts of lung tissue from IPF patients^26^. However, the latter study showed no images, nor reported uPA expression in epithelial cells. Lung fibroblasts dissociated and cultured from IPF lung biopsies are reported to express more uPA than lung fibroblasts of donors without evidence of lung disease^27^. In our study, concentrations of plasminogen at least an order of magnitude lower than those in plasma^28^ stimulated greater increases in plasmin and IL-6 production in IPF-derived lung fibroblasts than in those from controls. In contrast, plasmin mediated increases in IL-6 were not related to fibroblast donor status. These observations suggest that IPF-related increases in IL-6 production are a consequence of increased plasmin generation, rather than amplified plasmin signaling. Interestingly, plasminogen incubation also increased uPA production by lung fibroblasts of IPF donors, amplifying increased plasmin generation, whereas inhibiting uPA attenuated plasminogen activation and subsequent IL-6 production. The role of uPA in plasminogen-evoked IL-6 production is evidenced by the suppression of plasminogen activation and IL-6 production by the small molecule inhibitor UK122 and/or transfection with uPA gene-targeting RNA*i*.

Increased alveolar epithelial PAI-1 production in lung injury and disease contributes to the accumulation of airspace fibrin^7^,^27^. However, uPA levels are increased within the interstitium/fibrotic lesions of damaged parenchymal tissue^14^,^26^,^27^, potentially shifting the pericellular balance between uPA and PAI-1 to favour increased lesional plasmin production. In support of this hypothesis, bleomycin-induced lung injury increases localized plasminogen activation in the parenchymal tissue of mice, despite airspace fibrinolysis being suppressed^14^. Our data provides the first evidence that localized uPA production by IPF lung fibroblasts is associated with increased plasminogen activation. Lung fibroblast-generated plasmin not only stimulated increased fibrogenic IL-6 production, but also mitogenesis. Plasmin-evoked increases in proliferation were blocked by anti-IL-6 IgG, suggesting that autocrine IL-6 contributes to the proliferative effects of plasmin. Similarly, autocrine IL-6 mediates the proliferative effects of IL-1α on lung fibroblast proliferation^15^. Plasmin(ogen) has also been observed to elicit anti-fibrogenic responses in lung fibroblasts, including apoptosis^29^ and increased prostaglandin E_2_ (PGE_2_) production^30^, but only at concentrations much higher than the concentrations used in the current study. Moreover, plasminogen at 50 μg/mL causes apoptosis of embryo-derived cultured lung fibroblasts by proteolysis of secreted fibronectin, leading to cell detachment and anoikis^29^. The effect of higher concentrations of plasmin on cell adhesion (or potentially PGE_2_ production) may explain why plasmin(ogen) exhibited bell-shaped concentration-response curves for lung fibroblast mitogenesis. These distinct responses to plasminogen raise the possibility that chronic low levels of plasma leakage are fibrotic, whereas high levels of plasma leakage associated with extensive acute lung injury may serve to limit fibrogenesis.

The G protein-coupled receptor, PAR-1, is activated by proteases and implicated in many lung diseases. PAR-1 expression is increased in macrophages and fibroblasts in fibrotic lesions in IPF^31^,^32^. Inhibition of PAR-1 activation reduces lung inflammation and fibrosis in bleomycin-induced lung injury^31^,^32^. Furthermore, PAR-1 activation in lung fibroblasts elicits increased cytokine production, collagen expression and proliferation^31^,^32^,^33^. In the current study, plasmin(ogen)-stimulated IL-6 production by lung fibroblasts was attenuated by directly inhibiting PAR-1 using genetic (RNA*i),* immunological (neutralizing IgG) or pharmacological (SCH79797) inhibition of PAR-1, or indirectly by selective inhibition of MAPK kinases known to signal downstream of PAR-1^33^. Interestingly reducing PAR-1 expression also attenuated plasminogen activation. Thus, PAR-1 is likely to have a role in the increased uPA production that occurs with plasminogen and/or plasmin exposure.

In this study, cross-sectional data from IPF and non-IPF donors indicates that serum levels and activity of uPA are increased in IPF. Serum uPA levels/activity of IPF patients were inversely correlated with lung function as measured by %predicted FVC. Two molecular sizes of uPA were detected (~54 and 31 kDa), with the lower molecular weight protein being a proteolytic product of plasmin that retains uPA activity, but does not bind the uPA receptor, uPAR. Serum levels of uPA are an important diagnostic and prognostic marker for many forms of cancer^34^,^35^. However, our study is the first to show an association between IPF serum uPA levels or activity. Additional cross-sectional and longitudinal studies with a greater number and diversity (sex, disease stage and rate of progression, treatment history) of subjects will be needed to evaluate whether serum uPA may serve as a biomarker of IPF progression and treatment response. Interestingly, levels of uPA in the BALF of IPF patients have been reported to be lower or similar when compared to those of controls^9^,^10^. A decrease in uPA levels in IPF is possibly explained by a decrease in uPA (and increase in PAI-1) expression in the alveolar epithelium in the disease state^10^. It should also be noted that previous measurements of uPA in BALF were conducted using ELISA, which may not detect uPA when complexed with other proteins including PAI-1 and α2-macroglobulin. Complexes of uPA and α2-macroglobulin are detected at increased concentration in the lung tissue and alveolar edema fluid of patients with ARDS^36^. Higher levels of serum uPA in cancer are considered to be a consequence of spill over from increased uPA expression in tumour cells and the surrounding highly vascularised stroma. Higher levels/activity of uPA in sera of IPF patients may also be a result of spillover from the lung interstitium. However, further studies are required to ascertain whether increased uPA in IPF serum is of intra or extra-vascular origin.

**Figure 7:**
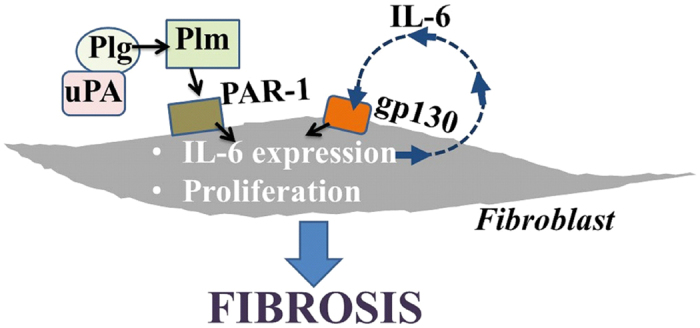
uPA mediates increased IL-6 production in lung injury and disease. uPA stimulates increased IL-6 production in lung fibroblasts by the regulation of plasmin (Plm) formation and activity, which perpetuates fibrosis in IPF.

In lung injury and disease, plasmin-evoked IL-6 production by lung fibroblasts involving uPA may contribute to tissue remodeling (Fig. 7). This proposed axis may be sufficient to drive the progression of fibrosis, intensifying altered IL-6-gp130-Stat3 signaling in IPF^23^. Considering its potential role in lung fibrosis, uPA may be a treatment target for IPF. As an emerging therapeutic target in oncology, highly selective small molecule inhibitors of uPA have already been investigated in pre-clinical and clinical cancer studies^6^.

In this study uPA was shown to mediate fibrogenic responses in lung fibroblasts including increased IL-6 production and proliferation. Furthermore, uPA inhibition reduced lung fibroblast IL-6 production and proliferation. Increased uPA expression and activity in the lung tissue and serum of IPF patients provides key evidence for validation of uPA as a drug target in IPF.

## Methods

### Human samples

Lung tissue and serum were collected from patients with confirmed IPF or controls (healthy donors). Diagnosis of IPF was made following multidisciplinary review as recommended in the ATS/ERS/JRS/ALAT Guidelines. All patients had provided written, informed consent and ethical approval was provided by The Alfred Hospital Human Research Ethics committee (#1002113 and #336/13), following guidelines from the National Health and Medical Research Council (Australia). See Table S1 of online supplement for patient characteristics of serum samples.

### Cell culture and enumeration

Lung fibroblast (LF) cell cultures were established from parenchymal tissue as described previously^37^ under local ethical approval from the University of Melbourne (HREC980168X), following guidelines from the National Health and Medical Research Council (Australia). For each experiment, primary cultures were established from tissue of separate patients, with cells grown in 6, 24 or 96 well plates in serum-containing Dulbecco’s Modified Eagles Medium (DMEM) at 37 °C (5% CO_2_). Donors were classified as either IPF or controls (lung transplant recipients or donors with no evidence of ILD). In mechanistic experiments (*e.g.* RNA*i* transfection), data from LFs of IPF and controls were combined. Cells were maintained in serum free-DMEM for 24 h before addition of human plasminogen or plasmin (Roche, Australia). After 48 h, cells were harvested by trypsin dissociation and enumerated with the aid of a hemocytometer. See online data supplement for more detail.

### Immunohistochemistry

Paraffin-embedded sections of parenchymal lung tissue were immunostained for uPA, IL-6 or α-smooth muscle actin (α-SMA), or histologically stained for collagen using Masson’s Trichrome. Antigen was identified by antibodies to uPA (rabbit, 1:100 dilution, Abcam, Cambridge, UK), IL-6 (mouse, 1:100 dilution; Abcam, Cambridge, UK) or α-SMA (rabbit, 1:250 dilution; Santa Cruz Biotechnology Inc, Dallas, TX, USA). Antibody staining was completed using the Dako EnVision anti-rabbit kit as appropriate (Dako Corp., Carpinteria, CA, USA) and 3,3′-diaminobenzidine (Sigma-Aldrich, St Louis, MO, USA). Sections were counterstained with hematoxylin.

### Measurement of plasmin activity, IL-6, PAI-1 and uPA

Following plasminogen incubation, plasmin activity in the supernatants of LFs was measured using a fluorogenic substrate^38^. Levels of human IL-6, plasminogen activator-1 (PAI-1) and uPA in LF supernatants were measured by specific sandwich enzyme-linked immunosorbent assays (ELISA) using commercial kits for IL-6 (BD Biosciences, CA, USA), PAI-1 (RnDSystems, MN, USA) and uPA (RnDSystems, MN, USA). Levels of uPA in human serum were detected by Western blotting as described in the online supplement. Activity of uPA in serum was detected using a fluorogenic substrate, as previously described^38^.

## RNA*i* transfection

Cells were seeded in 24 well plates in antibiotic-free serum containing DMEM and transfected 20 h later with 30 nM RNA interference (RNA*i*) duplex oligonucleotides using RNA*i*Max Lipofectamine (Invitrogen, CA, USA). Cells were incubated with Lipofectamine-RNA*i* complex for 6 h, before incubation in serum-free DMEM 20 h prior to plasmin(ogen) addition. uPA RNA*i* (Invitrogen, CA, USA) and PAR-1 and negative control RNA*i* (Genepharma, Shanghai, China) were used in the study. The following RNA*i* sequences were used: PAR-1, 5′-GGCAGUUGAUGGCAAGUAATT-3′; uPA, 5′-GCCCUCCUCUCCUCCAGAAGAAUUA-3′; and the negative control, 5′-UUCUCCGAACGUGUCACGUTT-3′.

### Western blot analysis

Lysates of cells grown in 24 well plates were prepared under reducing conditions, subjected to SDS polyacrylamide gel electrophoresis (SDS-PAGE) and electroblotted as described previously^39^. Serum samples were diluted 100 fold in PBS, before 10 μg of each sample was also subjected to SDS-PAGE as described previously^38^. For serum samples, all gels included one sample (a control subject) used as an internal reference for densitometric analysis. Serum samples from IPF and non-IPF were resolved on gels, electroblotted and immunostained in parallel. Following electroblotting, membranes were stained with Ponceau Red to verify uniform protein transfer. Ponceau red staining was also used in densitometric analysis to account for variation in loading between sera samples. Electroblotted membranes were blocked with 5% skim milk in TBS-T (10 mM Tris; 75 mM NaCl; 0.1% Tween-20; pH 7.4) for 1 h. Membranes were incubated overnight at 4 °C with either anti-PAR-1 antibody (ATAP2, mouse monoclonal; IgG, 1:500, Santa Cruz Biotechnology, USA); or anti-uPA (rabbit polyclonal IgG, 1:1000, Abcam, Cambridge, UK) diluted in 3% bovine serum albumin in TBS-T. Blots were washed three times with TBS-T prior to incubation with secondary antibody, goat anti-mouse (Chemicon) or anti-rabbit (Cell Signaling Technology) IgG-horse raddish peroxidase conjugate, diluted 1:5000 in 5% skim milk/TBS-T) for 1 h at room temperature. After three washes with TBS-T, antigen was detected by enhanced chemiluminescence (Amersham Biosciences, UK) using a Gel Doc XR imaging system (BioRad). Membranes of cell lysates were then stripped by incubation with 30 mL of 0.1 M glycine solution (pH 2.9) for 1 h at room temperature, blocked and incubated with anti-β-actin (mouse monoclonal antibody, 1:5000, Abcam, Cambridge, UK). Subsequent washes, secondary antibody incubation and chemiluminescence were as described above.

### Real-time polymerase chain reaction (PCR)

RNA extraction and Real-time PCR were conducted as previously described^37^. For more detail, see the online supplement.

### Statistical analysis

Data are presented as the mean ± SEM. For cell culture, *n* represents individual experiments, with each experiment being conducted using cells from a different donor. All data were statistically analyzed by two-way analysis of variance (ANOVA) with repeated measures (except where stated otherwise) and treatment groups compared with Bonferroni’s *post-hoc* tests (Graphpad Prism 5.0, Graphpad, San Diego, CA). A value of P < 0.05 was considered to be statistically significant.

## Additional Information

**How to cite this article**: Schuliga, M. *et al*. The fibrogenic actions of lung fibroblast-derived urokinase: a potential drug target in IPF. *Sci. Rep.* **7**, 41770; doi: 10.1038/srep41770 (2017).

**Publisher's note:** Springer Nature remains neutral with regard to jurisdictional claims in published maps and institutional affiliations.

## Supplementary Material

Supplementary Information

## Figures and Tables

**Table 1:** Plasmin stimulates increased IL-6 and uPA production by LFs.

Plasmin (mU/mL)	IL-6 (% basal)			uPA (% basal)		
	1.5	5	15	1.5	5	15
Control	136 ± 10	203 ± 40*	230 ± 30*	68 ± 14	85 ± 21	330 ± 169
IPF	148 ± 27	228 ± 36*	220 ± 33*	103 ± 26	691 ± 418	1042 ± 439*^

Levels of IL-6 and uPA in the culture supernatants of LFs from IPF (n = 6) and control (n = 5) donors incubated with plasmin for 24 h.*P < 0.05 compared to baseline. ^P < 0.05 compared to non-IPF at 15 mU/mL.
