# Supplementary material for: The fibrogenic actions of lung fibroblast-derived urokinase: a potential drug target in IPF
Source: Sci Rep. 2017 Jan 31;7:41770. doi: 10.1038/srep41770 (PMC5282574; doi:10.1038/srep41770)
Supplement: Supplementary Information [file srep41770-s1.pdf]

The fibrogenic actions of lung fibroblast-derived urokinase: a potential drug target in IPF

Michael Schuliga, Jade Jaffar, Trudi Harris, Darryl G Knight, Glen Westall & Alastair G Stewart

Online Supplement

Methods

IPF serum donor characteristics

ID	Gender	Age	BMI	Smoking history	FEV1 %predicted	FVC %predicted	TLCO %predicted
1	Male	63	24.8	ex 1995	57	48	32
2	Male	72	NA	NA	87	78	44
3	Male	60	24.3	never	78	69	45
4	Male	67	23.5	ex 2001	70	69	32
5	Male	78	22.8	never	83	77	45
6	Male	80	26.9	ex 1957	107	83	64
7	Male	60	27.4	minimal	67	63	43
8	Female	81	28.4	ex	116	103	81
9	Male	62	29.7	Ex-1998	86	74	69
10	Male	73	27.4	Ex-1998	63	61	25
11	Male	72	26.8	Ex-1979	57	46	31
12	Male	66	28.4	Ex - 1991	106	94	73
13	Female	60	33.9	Ex-2013	38	34	
14	Male	76	28.5	Ex-1977	94	76	62
15	Male	75	25.8	Ex-1975	95	89	49
16	Male	70	27.3	no	78	65	49
17	Male	69	33.8	Ex-1983	60	51	64
18	Male	70	33	Ex-1982	93	98	54
19	Female	58	28.6	Ex-2013	68	58	14

Table E1. Characteristics of serum IPF donors. *Characteristics include gender, age, smoking history, body mass index (BMI), forced expiratory volume in 1 second (FEV_1) %predicted, forced vital capacity (FVC) % predicted and transfer factor of the lung for carbon monoxide (TLCO)*

%predicted. Blood withdrawal for serum and pulmonary function tests were conducted in either 2014 or 2015. NA, data not available.

Cell culture

Human lung fibroblast (LF) cultures from controls (Ctrl-LFs) were established using parenchyma obtained from macroscopically normal resections of lung transplant patients or donors (Alfred Hospital, Prahran, Melbourne) as described previously (1). Approval for receiving tissue was granted by the University of Melbourne's Human Research Ethics Committee (HREC980168X). LFs of IPF donors were established and obtained from the Alfred Lung Fibrosis Biobank (Alfred Hospital, Prahran, Melbourne) (2) or were a generous gift of Prof Judith Black (University of Sydney, NSW, Australia). A minimum of three separate cultures established from separate patients were used for each experiment, with cells between the 4th and 10th passage. Cells were seeded onto 6, 24 or 96 well plates (2×10^4 cells/cm^2) in Dulbecco's Modified Eagles Medium (DMEM) supplemented with L-glutamine (2 mM), sodium pyruvate (1 mM), non-essential amino acids (1% v/v, Sigma) and heat-inactivated fetal calf serum (5% v/v) and incubated at 37°C in air containing 5% CO_2. One day after seeding, the medium was removed and the cells were then incubated in serum free-DMEM containing bovine serum albumin (0.25% w/v) and supplements (L-glutamine, sodium pyruvate and non-essential amino acids) for a further 24 h before the addition of human plasminogen (0.5-50 μg/mL, Roche) or human plasmin (0.5-50 mU/mL, Roche). After an additional 24 h, culture supernatants were collected for assay of IL-6. In selected experiments, pharmacological inhibitors were added to cell culture medium at a final concentration of 10 μM, 30 min before the addition of plasmin(ogen). The final concentration of DMSO, the diluent for these inhibitors, was 0.1 % v/v, and all cells were exposed to the same concentration of DMSO. The inhibitors used were: UK122 for uPA; LY294002 for PI3K/Akt; PD98059 for ERK1/2; and SB203580 for p38 MAPK. In selected experiments, anti-PAR-1 (ATAP2, Santa Cruz Biotechnology, USA), anti-IL-6 (BD Biosciences, CA, USA) or control IgGs (Santa Cruz Biotechnology, USA) were used at 2 μg/mL.

Cell enumeration

After 48 h incubation with plasmin(ogen), attached cells in 24 well plates were dissociated and harvested by incubation with trypsin (0.125% w/v) and EDTA (0.02% w/v) in PBS. For selected experiments, detached cells in the culture medium were pelleted by centrifugation. Cells were resuspended in 0.25% v/v BSA in PBS containing trypan blue (0.2% w/v) and viable cells counted (in duplicate) with the aid of a hemocytometer.

RNA extraction and real-time polymerase chain reaction (PCR)

RNA was purified from cells maintained in either 24 or 96 well culture plates using Trizol (Invitrogen), according to the manufacturer's instructions. RNA was extracted from lung tissue using Qiagen mini prep columns (Qiagen), according to the manufacturer's instructions. Before RNA extraction, frozen tissue was crushed using a mortar and pestle in liquid N_2 to prevent thawing. Reverse transcription of total RNA and the subsequent real-time polymerase chain reaction using an ABI Prism 7900HT sequence detection system (Applied Biosystems) with the relevant forward and reverse primers were conducted as previously described (1). The following primers were used: Human PAR-1, 5'-CAA ATG CCA CCT TAG ATC CCC-3' (sense) and 5'- CTT CTG AGA TGA ATG CAG GAA GT-3' (antisense); human uPA, 5'-GAG GGC AGC ACT GTG AAA TAG ATA-3' (sense) and 5'-CCA TTC CCC TCA TAG CAG GTT-3' (antisense); human CCND1, 5'-ACT ACC GCC TCA CAC GCT TC -3' (sense) and 5'-CAG TCT GGG TCA CAC TTG ATC AC-3' (antisense); human 18S ribosomal RNA (18S rRNA) 5'-CGC CGC TAG AGG TGA AAT TC-3' (sense) and 5'-TTG GCA AAT GCT TTC GCT C-3' (antisense). The threshold cycle (CT) value determined for each gene of each sample was normalized against that obtained for 18S rRNA, which was included as internal control. For each sample, the level of mRNA for a particular gene is proportional to $2^{-(\Delta CT)}$, where ΔCT is equal to the CT value of the target gene minus the CT value of 18S rRNA.

Figure legends

Figure S1. **uPA, collagen and α-SMA in lung tissue of IPF patients.** Parallel sections of parenchymal tissue from three separate IPF patients (ALF008, ALF016 and ALF019) stained for uPA (left panel), collagen using Masson's trichrome (middle panel) and α-smooth muscle actin (α-SMA) (right panel). The scale bars in the 40 X and 200 X images are 500 and 100 micron respectively.

Figure S2. **uPA, collagen and α-SMA in lung tissue of controls.** Parallel sections of parenchymal tissue from three separate controls (ALF012, ALF013 and ALF017) stained for uPA, collagen using Masson's trichrome, and α-smooth muscle actin (α-SMA). Sections were histologically processed in parallel with the IPF tissue presented in **Fig S1**. The scale bars in the 40 X and 200 X images are 500 and 100 micron respectively.

Figure S3. **Serum uPA activity is increased in IPF.** (a) uPA enzyme activity in sera from IPF patients and controls (*$P<0.05$). (b) The relationship between uPA activity of IPF patients and lung function (FVC %predicted). The linear line is the regression line, whereas the top and bottom curved lines represent the 95% confidence intervals. Data (b) were analyzed using the Pearson's correlation test, with r and P (one-tailed) values provided.

Figure S4. **No correlation between serum levels of uPA and age.** The relationship between serum (a) uPA levels or (b) uPA activity and donor age. Data was analyzed using Pearson's correlation test. For IPF patients, r values are (a) -0.05 and (b) 0.03, whereas the P values (one-tailed) are (a) 0.43 and (b) 0.46.

Figure S5. **PAI-1 levels of control and IPF sera are similar.** (a) The levels of PAI-1 in serum as measured by ELISA. (b-c) The relationship between serum PAI-1 levels and (b) FVC (%predicted) and (c) uPA levels of IPF patients.

Figure S6. **siRNA transfection reduces uPA and PAR-1 expression.** Levels of (a) uPA and (b) PAR-1 mRNA in cultures of RNA*i*-transfected LFs. Data analyzed by student's t test. **P<0.01 (n=3).

Figure S7. **UK122, a uPA inhibitor, attenuates plasminogen activation.** The effect of UK122 (10 μM) on plasmin activity in the conditioned medium of LFs following incubation with plasminogen (15 μg/mL) for 3 h. Data was analysed by Wilcoxon matched pairs signed-rank test (P<0.05).

Figure S8. **PAR-1 mediates plasmin-stimulated IL-6 production**. Levels of IL-6 in the supernatant of LFs incubated with plasmin (5 mU/mL) for 24 h following (a) RNA*i* transfection or (b) co-incubation with IgG (2 μg/mL). **P<0.01 (n=4).

Figure S9. **Plasmin-stimulated IL-6 production involves MAPK signaling**. Levels of IL-6 in the supernatant of LFs incubated with plasmin (5 mU/mL) for 24 h. Cells were pre-treated with the kinase inhibitors, LY294002 (LY, 10 μM), PD98059 (PD, 10 μM) or SB203580 (SB, 10 μM). *P<0.05, **P<0.01 (n=5).

Figure S10. **Plasminogen and plasmin increase cell detachment.** The number of attached and non-attached cells (%control, total cells) in cultures of LF cells following incubation with either plasminogen or plasmin for 48 h. *P<0.05, **P<0.01 versus control (n=4-6). For all treatments, the percentage of attached cells that were non-viable was negligible.

Figure S11. **Plasmin stimulates increased cell cyclin D1 expression in a PAR-1-dependent manner.** Levels of cell cyclin D1 (CCND1) mRNA following RNA*i* transfection and subsequent

plasmin incubation for 6 h. *P<0.05 (n=6)

Figure S1

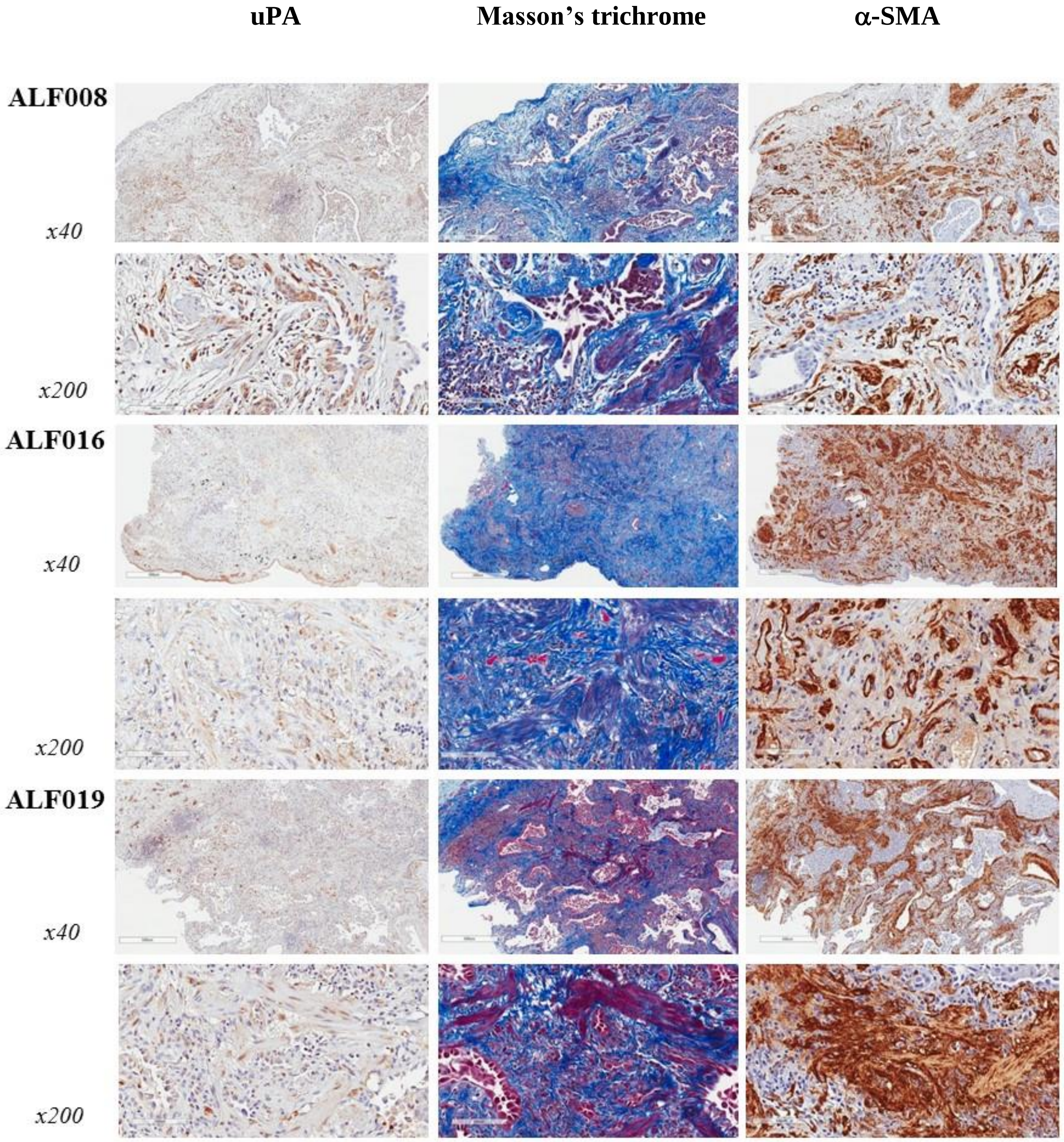

uPA
Masson's trichrome
α-SMA
ALF008
x40
x200
ALF016
x40
x200
ALF019
x40
x200

Figure S2

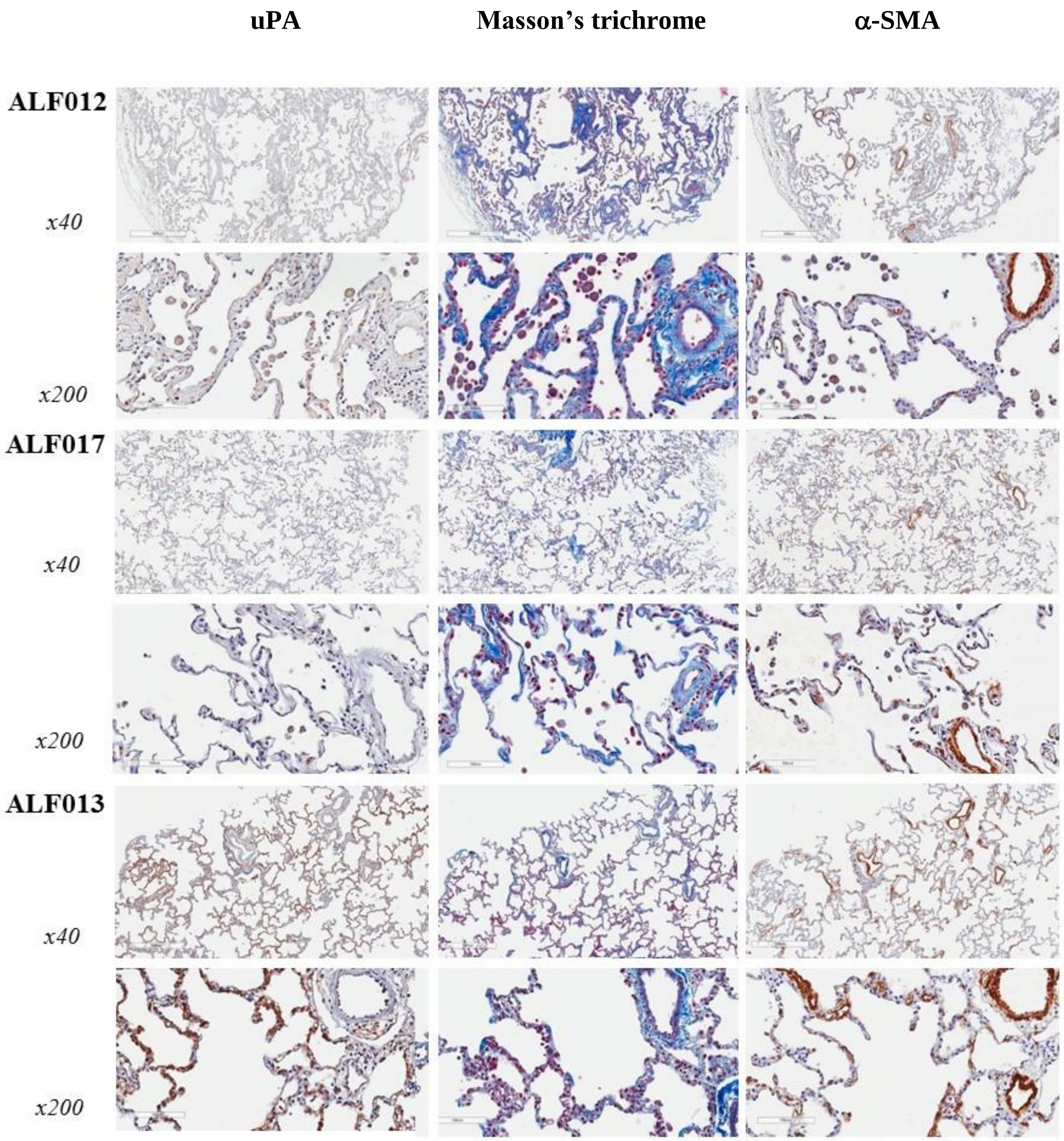
uPA
Masson's trichrome
α-SMA
ALF012
x40
x200
ALF017
x40
x200
ALF013
x40
x200

Figure S3

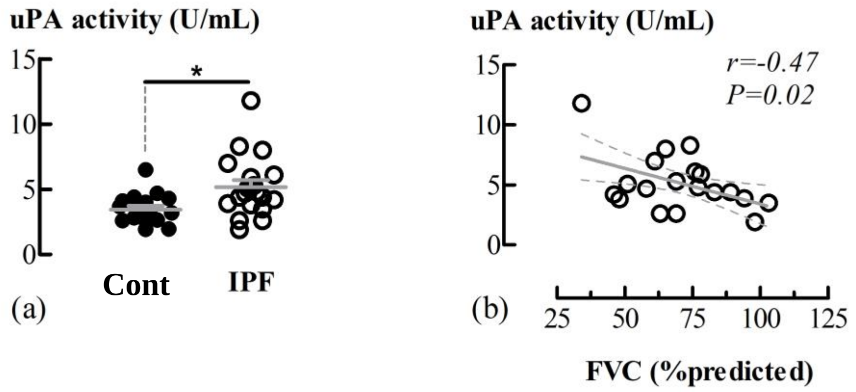
uPA activity (U/mL)
15
10
5
0
*
Cont
IPF
(a)
uPA activity (U/mL)
r=-0.47
P=0.02
15
10
5
0
(b)
25
50
75
100
125
FVC (%predicted)

Figure S4

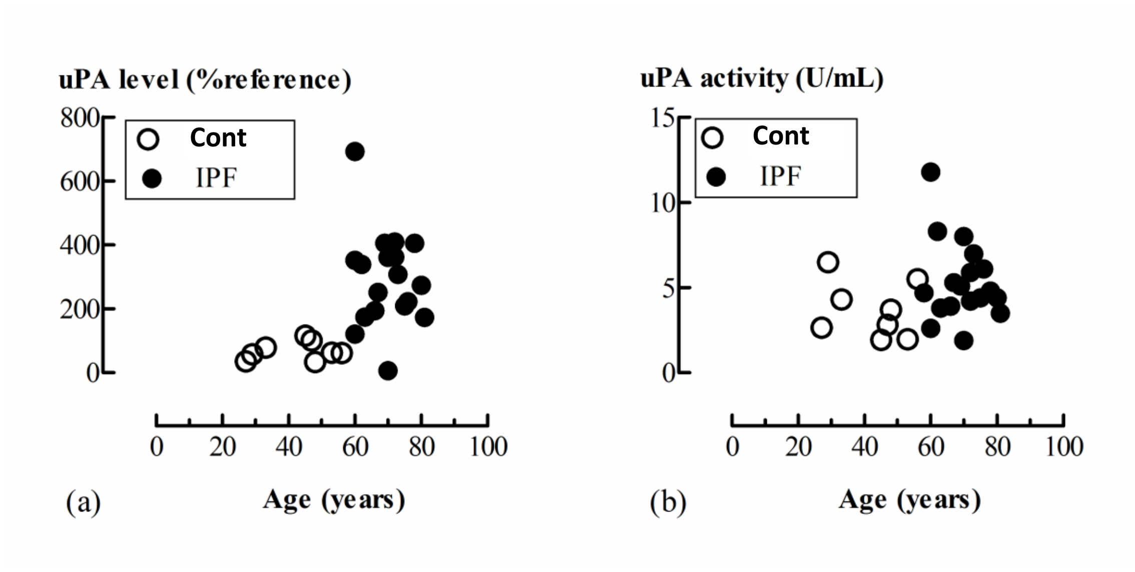

uPA level (%reference)
800
600
400
200
0
Cont
IPF
0
20
40
60
80
100
(a)
Age (years)
uPA activity (U/mL)
15
10
5
0
Cont
IPF
0
20
40
60
80
100
(b)
Age (years)

Figure S5

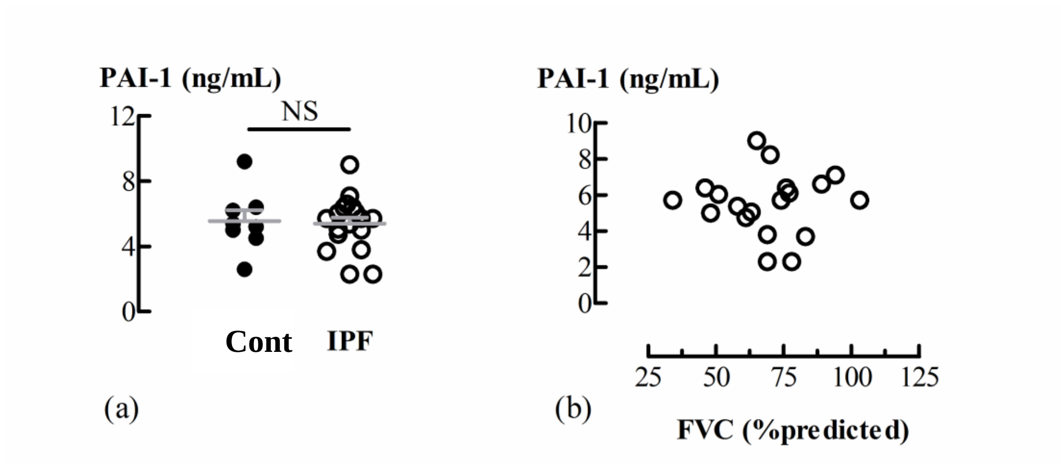

PAI-1 (ng/mL)
12
8
4
0
NS
Cont
IPF
(a)
PAI-1 (ng/mL)
10
8
6
4
2
0
25
50
75
100
125
(b)
FVC (%predicted)

Figure S6

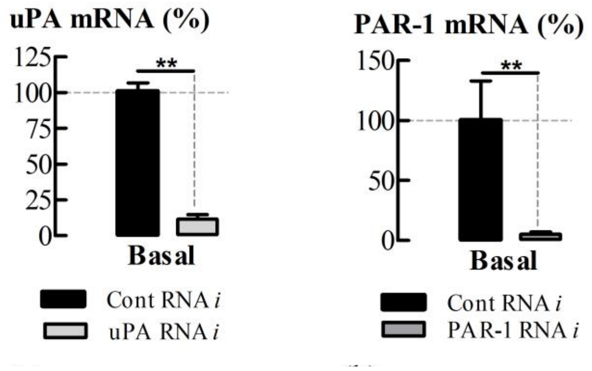
uPA mRNA (%)
125
100
75
50
25
0
**
Basal
Cont RNA *i*
uPA RNA *i*
PAR-1 mRNA (%)
150
100
50
0
**
Basal
Cont RNA *i*
PAR-1 RNA *i*

Figure S7

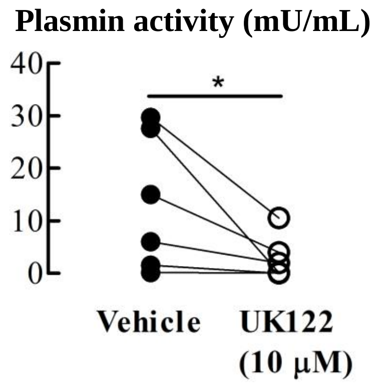

Figure S8

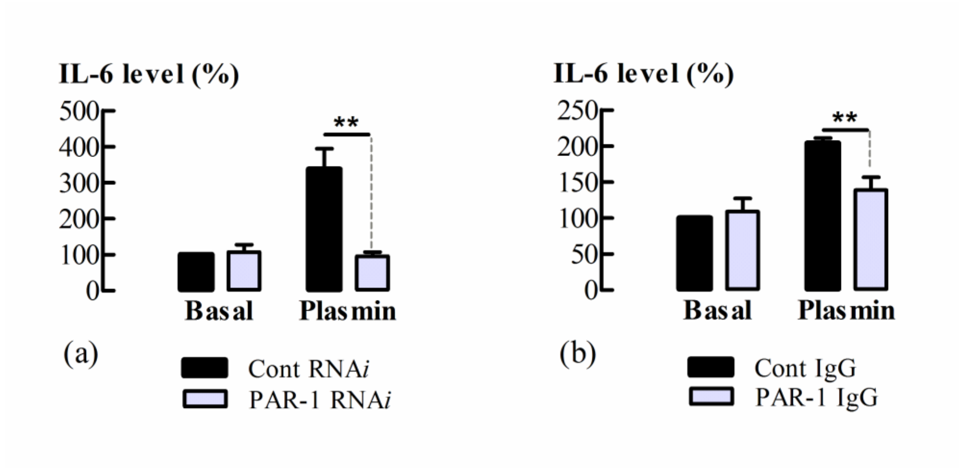
IL-6 level (%)
500
400
300
200
100
0
**
Basal
Plasmin
(a)
Cont RNAi
PAR-1 RNAi
IL-6 level (%)
250
200
150
100
50
0
**
Basal
Plasmin
(b)
Cont IgG
PAR-1 IgG

Figure S9

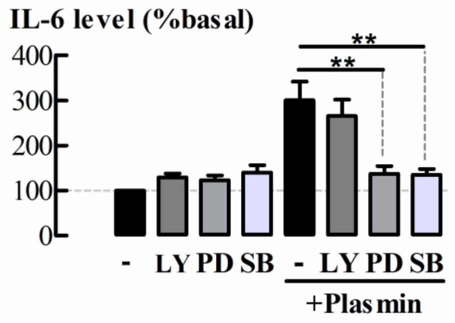

Figure S10

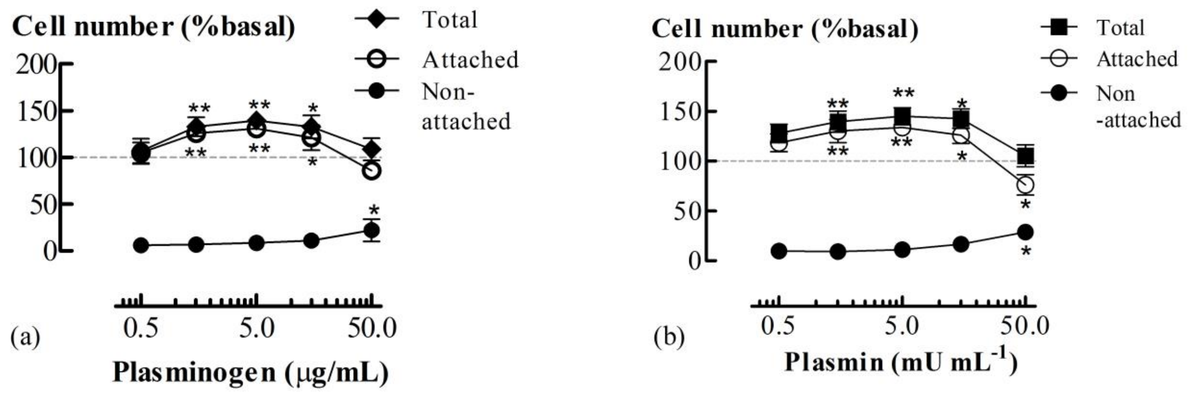

Cell number (%basal)
200
150
100
50
0
Total
Attached
Non-attached
0.5
5.0
50.0
(a)
Plasminogen (μg/mL)
Cell number (%basal)
200
150
100
50
0
Total
Attached
Non -attached
0.5
5.0
50.0
(b)
Plasmin (mU mL-1)

Figure S11

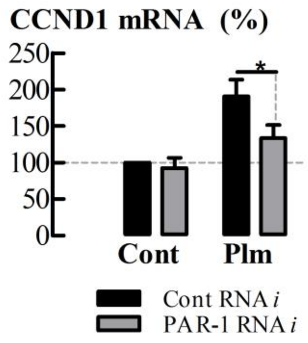

References

1. Schuliga MJ, See I, Ong SC, Soon L, Camoretti-Mercado B, Harris T, Stewart AG. 2009. Fibrillar collagen clamps lung mesenchymal cells in a nonproliferative and noncontractile phenotype. *Am J Respir Cell Mol Biol* 41: 731-41
2. Jaffar J, Unger S, Corte TJ, Keller M, Wolters PJ, Richeldi L, Cerri S, Prele CM, Hansbro PM, Argraves WS, Oliver RA, Oliver BG, Black JL, Burgess JK. 2014. Fibulin-1 predicts disease progression in patients with idiopathic pulmonary fibrosis. *Chest* 146: 1055-63
